# Carbon Ion Beam Radiotherapy for Sinonasal Malignant Tumors Invading Skull Base

**DOI:** 10.1155/2014/241856

**Published:** 2014-06-09

**Authors:** Nobuo Ohta, Yusuke Suzuki, Azusa Hasegawa, Masaru Aoyagi, Seiji Kakehata

**Affiliations:** ^1^Department of Otolaryngology, Head and Neck Surgery, Faculty of Medicine, Yamagata University, 2-2-2 Iida-nishi, Yamagata 990-9585, Japan; ^2^Research Center for Charged Particle Therapy, National Institute of Radiological Sciences, Chiba 263-8555, Japan

## Abstract

*Objective*. To evaluate the treatment outcome and prognostic factors in patients with sinonasal malignant tumors invading skull base. *Study Design and Setting*. A retrospective clinical study at the Yamagata University School of Medicine. *Subjects and Methods*. Three patients with sinonasal malignant tumors invading skull base were presented in present study. All patients were treated with carbon ion beam radiotherapy. The prescribed dose to the center of the clinical target volume was 64.0 GyE/16 fractions over 4 weeks at 4.0 GyE/fraction per day. *Results*. All patients completed carbon ion beam radiotherapy without an interval. The mean observation period was 39.6 months (range: 11–54 months). There were no local or regional recurrences in all cases; however, one patient had a metastasis in distant organs. Regarding the complications, visual loss was observed in one eye of one patient whose optic nerve was entirely involved by the tumor and field of carbon ion beam radiotherapy. Radiation induced brain injury was observed in two patients; however, these patients do not complain about neurological abnormality and had no treatment for radiation induced brain necrosis. *Conclusions*. Carbon ion beam radiotherapy for sinonasal malignant tumors invading the skull base showed therapeutic effectiveness.

## 1. Introduction

In thetreatmentofsinonasal malignant tumors invading skullbase,criticalorganssuchascranialnerves,eyes,brainstem,cochlea, and brain tissues limit the application of complete surgical resection procedures and high-dose irradiation to the target region. Due to the dose limitation in the skull base region, local control rates have been very poor in the past [1–3]. Carbon ion beam radiotherapy has been developed to overcome these clinical problems and applied as the treatment modality, and it offers superior dose conformity in the treatment of locally advanced malignant tumors compared with conventional radiotherapy. Little is known about the outcomes and prognostic factors in patients with sinonasal malignant tumors invading skull base. In present study, three cases of sinonasal malignant tumors invading the skull base were presented and diagnostic and therapeutic options are discussed.

## 2. Subjects and Methods

Three patients with locally advanced malignant sinonasal tumors extending to the skull base were treated with carbon ion radiotherapy. All patients were not indicated for curative or declined surgery. Three representative cases are presented here.

### 2.1. Clinical Histories

#### 2.1.1. Case 1

**Figure 1:**
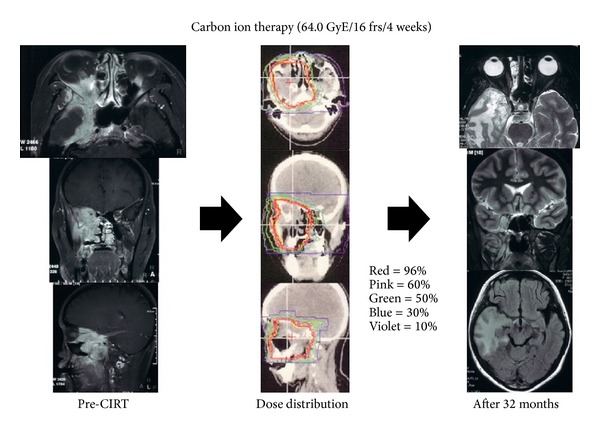
A case of 43-year-old Japanese female with adenoid cystic carcinoma in skull base. Axial, coronal, and sagittal contrast T1-weighted magnetic resonance image before carbon ion beam radiotherapy shows marginal enhancement and surrounding edema; dose distribution of carbon ion radiotherapy in axial CT image and axial contrast-enhanced T1-weighted magnetic resonance image 32 months after carbon ion beam radiotherapy show enhancement and edema in the right temporal lobe. This patient received no treatment for cerebral radiation injury. CIRT: carbon ion beam radiotherapy.

A 43-year-old Japanese female developed her right exophthalmos and slowly progressive deterioration of right visual acuity (0.2) in September, 2008. The ophthalmologist had pointed out the sinonasal tumor and the patient was referred to our department. CT and MRI showed the sinonasal tumor extending to skull base and right orbit (Figure 1). Endoscopic surgery was performed and the pathological diagnosis was adenoid cystic carcinoma. There was no indication of curative surgery and carbon ion beam radiotherapy was performed. The tumor gradually shrank, and a complete response was observed 22 months after the therapy. Her left visual acuity began to increase and recovered to 1.0 in 8 months after the therapy. The tumor was still controlled 54 months after the carbon ion beam radiotherapy. Neurological findings were not observed in spite of relatively spacious temporal lobe brain necrosis.

#### 2.1.2. Case 2

**Figure 2:**
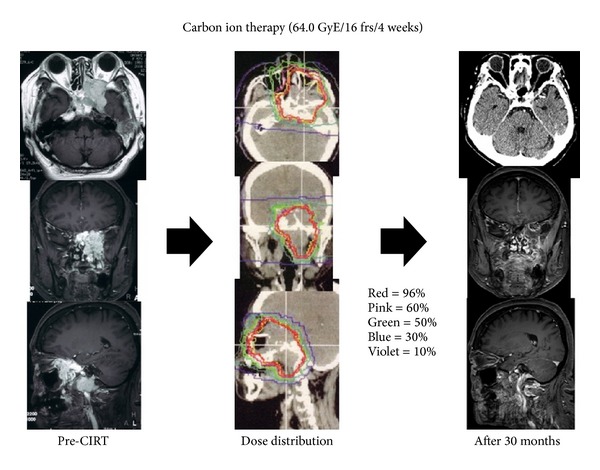
A 43-year-old Japanese female with adenoid cystic carcinoma in skull base. Axial, coronal, and sagittal contrast T1-weighted magnetic resonance image before carbon ion beam radiotherapy, dose distribution of carbon ion radiotherapy in axial CT image, and axial contrast-enhanced T1-wighted magnetic resonance image 30 months after carbon ion radiotherapy show enhancement and edema. This patient received no treatment for cerebral radiation injury. CIRT: carbon ion beam radiotherapy.

A 57-year-old female had double vision and deterioration of visual acuity (20 cm/counting fingers) gradually in September, 2008. The tumor was detected at previous hospital and the patient was referred to our department for further examination and treatment (Figure 2). Endoscopic surgery was performed and the pathological diagnosis was adenoid cystic carcinoma. There was no indication of curative surgery and carbon ion beam radiotherapy was performed. The tumor gradually shrank, and a complete response was observed 22 months after the therapy. Her left visual acuity continued to decrease and total visual loss was observed in 24 months after the therapy. The local site was still successfully controlled 54 months after the therapy. Neurological findings were not observed in spite of relatively spacious temporal lobe brain injury.

#### 2.1.3. Case 3

A 41-year-old male developed headache and frontal swelling in November, 2010. The large frontal sinus tumor invading the skull base was detected by CT and MRI. Endoscopic surgery was performed and pathological diagnosis was squamous cell carcinoma. Carbon ion beam radiotherapy was performed. The primary site was successfully controlled; however, multiple lung and bone metastases were detected 8 months after the therapy. He was dead by 11 months after the treatment.

*Carbon Ion Beam Radiotherapy*. Determination of gross target volume was calculated based on contrast-enhanced CT and MRI. Irradiation was performed once per day for 4 days per week with carbon ion beams. Doses of carbon ions were expressed in photon equivalent doses (GyE). The prescribed dose to the center of the clinical target volume was 64.0 GyE/16 fractions over 4 weeks at 4.0 GyE/fraction per day based on previous clinical trials for head and neck cancers including malignant melanoma, adenocarcinoma, adenoid cystic carcinoma, and squamous cell carcinoma [2, 3]. The planning target volume included margins of 3.0–5.0 mm around the clinical target volume, and this could be modified manually. Vital organs including eyeball(s), optic nerve, optic chiasm, cochlea, and brain stem were outlined on the planning CT images to permit dose-volume histogram analysis and tried to avoid the damage to these tissues as much as possible, respectively.

*Followup*.All patients were regularly followed up by CT or MRI every 1 or 2 months for the first 6 months after carbon ion beam radiotherapy and thereafter every 3 to 6 months.

## 3. Results

All patients completed carbon ion beam radiotherapy without an interval. The mean observation period was 39.6 months (range: 11–54 months). There were no local or regional recurrences in all cases; however, one patient had a metastasis in distant organs. Regarding the complications, visual loss was observed in one eye of one patient whose optic nerve was entirely involved by the tumor and field of carbon ion beam radiotherapy. Radiation induced brain injury was also observed in two patients; however, these patients do not complain about neurological abnormality and had no treatment for radiation induced brain necrosis.

## 4. Discussion

The generally accepted treatment for sinonasal malignant tumors is surgical resection. In the treatment of a sinonasal malignant tumors invading skull base, complete resection is rarely possible, and carbon ion beam radiotherapy offers superior dose conformity compared with conventional X-ray therapy [1–13]. In addition, carbon ion beam radiotherapy has a higher relative biological effectiveness compared with protons or X-ray beams. It has been widely accepted that, in the treatment of skull base tumors, critical organs such as cranial nerves, eyes, optic nerves, cochlea, brain stem, and brain tissue limit the application of high-dose irradiation to the target lesion. It is reported that a dose fractionation of 60.8 Gy/16 factions for 4 weeks was decided as the recommended dose because of acceptable normal tissue reactions and good local tumor control [4].

Schardt et al. reported the clinical results of carbon ion beam radiotherapy in 236 patients with head and neck cancers [2]. 90% of the patients had locally advanced disease (T3, T4, local recurrence, or residual disease after surgery); the 5-year local control rate by histological type was 75% for the 85 patients with malignant melanoma, 73% for the 69 patients with adenoid cystic carcinoma, 73% for the 27 with adenocarcinoma, 61% for the 13 with papillary adenocarcinoma, and 61% for the 12 with squamous cell carcinoma. The 5-year overall survival rate was 68% for adenoid cystic carcinoma, 56% for adenocarcinoma, 35% for malignant melanoma, and 17% for squamous cell carcinoma.

With regard to complications of carbon ion beam radiotherapy, brain injury, vision loss, cataract, meningitis, CSF leakage, severe unilateral retinopathy, and facial nerve palsy have been reported previously [1–13]. For the treatment of sinonasal malignant tumors extending to the skull base, carbon ion beam radiotherapy has been used with the goal of preserving function or for cosmetic advantages. Preservation of visual acuity is crucial for patients' quality of life after treatment. Radiation induced brain injury is classified as an acute, early delayed, or late reaction according to its timing after radiotherapy [9–13]. Acute injury occurs during or just after completion of radiation therapy; early delayed injury develops few weeks (up to about 12 weeks) after radiation therapy [9–14]. Late reaction is one of the most serious complications of radiation therapy of head and neck tumors and develops after few months to several years after radiation therapy [9–14]. The spectrum of late radiation injury ranges from faint and limited damage to white matter to complete ischemic necrosis. Radiation induced brain necrosis is thought generally to be progressive and irreversible. In present report, carbon ion beam radiotherapy shows excellent results, with no local recurrence; however, brain injury and visual disturbance were observed. The study on the acceptable dose of carbon ion beam radiotherapy should be performed to prevent serious complications. We should keep in mind that carbon ion radiotherapy shows very good response to locally advanced malignant tumors even in limited cases and furthermore larger clinical trial consisting of carbon ion radiotherapy with existing or developing cancer therapy will be required.

## 5. Conclusions

Our results suggest that carbon ion beam radiotherapy is highly effective for locally advanced malignant tumor extending to skull base in even few and limited cases; however, distant metastasis should be kept in mind for management of these patients.
